# A Comparative Analysis for 2D Object Recognition: A Case Study with Tactode Puzzle-Like Tiles

**DOI:** 10.3390/jimaging7040065

**Published:** 2021-04-01

**Authors:** Daniel Silva, Armando Sousa, Valter Costa

**Affiliations:** 1 Institute for Systems and Computer Engineering, Technology and Science (INESC TEC), 4200-465 Porto, Portugal; asousa@fe.up.pt; 2 Department of Engineering, University of Trás-os-Montes e Alto Douro (UTAD), 5000-801 Vila Real, Portugal; 3 Faculty of Engineering, University of Porto (FEUP), 4200-465 Porto, Portugal; 4 Institute of Science and Innovation in Mechanical and Industrial Engineering (INEGI), 4200-465 Porto, Portugal; ee09115@gmail.com

**Keywords:** computer vision, machine learning, 2D object recognition, HOG, SVM, VGG, ResNet, MobileNet, YOLO, SSD

## Abstract

Object recognition represents the ability of a system to identify objects, humans or animals in images. Within this domain, this work presents a comparative analysis among different classification methods aiming at Tactode tile recognition. The covered methods include: (i) machine learning with HOG and SVM; (ii) deep learning with CNNs such as VGG16, VGG19, ResNet152, MobileNetV2, SSD and YOLOv4; (iii) matching of handcrafted features with SIFT, SURF, BRISK and ORB; and (iv) template matching. A dataset was created to train learning-based methods (i and ii), and with respect to the other methods (iii and iv), a template dataset was used. To evaluate the performance of the recognition methods, two test datasets were built: *tactode_small* and *tactode_big*, which consisted of 288 and 12,000 images, holding 2784 and 96,000 regions of interest for classification, respectively. SSD and YOLOv4 were the worst methods for their domain, whereas ResNet152 and MobileNetV2 showed that they were strong recognition methods. SURF, ORB and BRISK demonstrated great recognition performance, while SIFT was the worst of this type of method. The methods based on template matching attained reasonable recognition results, falling behind most other methods. The top three methods of this study were: VGG16 with an accuracy of 99.96% and 99.95% for *tactode_small* and *tactode_big*, respectively; VGG19 with an accuracy of 99.96% and 99.68% for the same datasets; and HOG and SVM, which reached an accuracy of 99.93% for *tactode_small* and 99.86% for *tactode_big*, while at the same time presenting average execution times of 0.323 s and 0.232 s on the respective datasets, being the fastest method overall. This work demonstrated that VGG16 was the best choice for this case study, since it minimised the misclassifications for both test datasets.

## 1. Introduction

**Figure 1:**
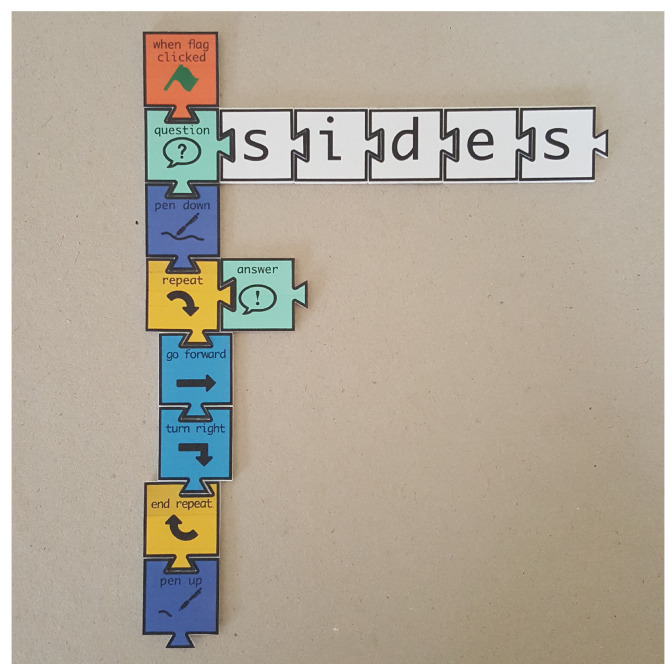
Tactode code example with 14 tiles that draws a regular polygon with the number of sides specified by the user.

Tactode [1,2,3,4] is a tangible programming system whose name comes from “tactile coding”. The main goal of Tactode is to bring robotics and programming closer to children in a more interactive and understandable way, while developing their programming skills and computational thinking [5]. This system is made up of a web application, tangible tiles, a simulator and a real robot. This way, the children create a code using the tiles, take a photograph of it and upload it to the web application, where they can test the tangible code with the simulator, to be further executed on a real robot [5]. Figure 1 shows an example of a Tactode code that draws a regular polygon and that can be interpreted as the following: after receiving a flag signal, the robot asks the user about the number of sides the polygon must have; when the user answers the question, the pen goes down to the correct position for drawing, and after, the robot enters a cycle where it goes forward and then turns right, drawing the sides of the polygon—this is repeated an amount of times that corresponds to the number of sides answered by the user; after completion of the drawing, the pen returns to the original position.

In this work, an image processing pipeline was used within a case study conducted on 32 Tactode tiles. The pipeline received an image and, after some segmentation steps, was capable of separating every tile present in the image to be further identified. The purpose of this work was to make a comparative analysis of the recognition performance of several methods used by the pipeline. The recognition methods were evaluated on two datasets that were created for testing. Furthermore, two training datasets were built to train distinct classifiers. All the datasets were automatically annotated and made available online. Although this work only focused on Tactode tiles, this study can most likely be extended to other types of tiles or similar 2D objects.

The main contribution of this work was the comparative analysis of a large number (12) of recognition methods in the specific task of recognising puzzle-like tiles. Additional contributions also included the dissemination of the code, the trained models and the datasets of this comparative study.

The remainder of this paper is organised as follows. Section 2 revises some methods for recognition tasks, and Section 3 introduces the selected approach and the methods used in this work. Section 4 presents the datasets that were built to test and train some of the methods, as well as the metrics utilised to evaluate their performance. The results acquired from this work are shown in Section 5 and are discussed later in Section 6. Lastly, Section 7 draws conclusions about this work.

## 2. Literature Review

In this section, the state-of-the-art for 2D object recognition is presented, focusing on some methods inserted in the domains of machine learning, matching of handcrafted features and template matching.

Template matching [6] is a method capable of classifying images by calculating a similarity measure between some templates and the target image. Thus, the template that presents the greatest similitude classifies the target image or locates itself in the image [7].

Regarding the algorithms for feature detection and description, one that must be paid attention to is HOG, since it is a well-known method that, when paired with an SVM, can form reliable classification models. HOG is a feature descriptor that regionally describes an image through normalised histograms of oriented gradients. In 2005, an implementation of the aforementioned combination (HOG and SVM) was designed to perform pedestrian detection [8], which turned out to be an innovative and successful approach. Moreover, other algorithms like SIFT, SURF, BRISK and ORB can be used for classification-oriented purposes when combined with a descriptor matcher. SIFT [9] is a feature detector and descriptor algorithm that detects keypoints and checks whether the keypoints are stable enough to be considered true interest points (otherwise, they are rejected). Then, orientation invariance is assigned to the true keypoints, and the descriptor is constructed by computing the gradient orientation and magnitude in the region around the keypoint location [10]. SURF has an implementation similar to SIFT, but it uses integral images that, along with box filters, help improve its performance. The descriptor is computed by the use of Haar wavelets and is invariant to scale and rotation; however it is less invariant to affine changes [11]. Another algorithm known for keypoint detection and description is BRISK, which uses the AGAST [12] method to perform keypoint detection, and the description phase consists of the determination of each keypoint direction and the aggregation of the results obtained from brightness comparison tests [13]. Another important algorithm to be mentioned is ORB. This is also a feature detection and description algorithm that makes use of FAST [14] and BRIEF [15] as a keypoint detector and descriptor, respectively. In this algorithm, the keypoint detection step is based on a variant of FAST with an orientation component, and the description stage utilises a rotation-aware BRIEF, resulting in a rotation and not-scale invariant descriptor [16].

Currently, the cutting edge methods for recognition tasks are based on machine learning. This domain is a subset of artificial intelligence whose objective is to teach a machine to learn from input data [17]. There are several types of learning that differ from each other regarding the purpose of the data and the way that they are handled: supervised learning was the one used in this work, defined by training a model with labelled data, and when tested, it was expected that the model would predict a labelled output. One of the many supervised learning methods is SVM, which maps input vectors into a dimensional space where a linear surface is built to improve the classes’ generalisation [18]. This method was primarily designed to address binary classification problems, but currently, SVM can also deal with multiclass challenges. For such tasks, there are some implementations: the one called “one-against-one” is the most competitive [19].

In addition to SVM, there are other methods, targeting recognition purposes with multiple classes, whose the models’ foundations are centred on a Convolutional Neural Network (CNN). These methods are inserted in a machine learning field called deep learning. In this work, we used methods of this type to attempt to identify objects in images; nonetheless, they are distributed in two distinct groups: object detection—the model detects and classifies the object(s) of the input image; and object classification—the only focus of the model is to classify the object present in the image.

Single-Shot Multibox Detector (SSD) and You Only Look Once (YOLO) are two of the main strategies concerning object detection. YOLO starts by resizing the input image to 448×448 and then divides it into a grid of S×S, where each cell is responsible for predicting one object [20]. For each cell, YOLO predicts *B* bounding boxes (each of them with a confidence score) and *C* class probabilities for each bounding box. The resulting prediction tensor is defined by an S×S×(B×5+C) shape [20]. The main benefits of this system over other object detection systems are its capability for real-time processing (with Graphical Processing Units (GPUs)) and for predicting the locations of objects and their classes using a single network [20]. Officially, there are three new versions of YOLO: the second version (YOLOv2) appeared in 2017 with improvements related to the accuracy and speed of detection and is also called YOLO9000 because it can detect in real-time more than 9000 distinct object categories [21]; the third version (YOLOv3) has even better performance regarding detection speed [22]; and YOLOv4 (the last official version of YOLO) is currently the fastest and most accurate detector [23] and follows a structure that has a CSPDarknet53 [24] backbone, a neck composed by SPP [25] and PAN [26], and the head corresponds to YOLOv3. SSD is another method to detect objects in images that relies on convolutional neural networks and is composed by a base network, responsible for image classification, followed by an extra network that provides detections at multiple scales. In [27], VGG16 was utilised as the base network of the model.

When the objective is to classify an object inside an image, methods like VGG16, VGG19, ResNet and MobileNet tend to be chosen, as they were designed for this specific goal. VGG16 and VGG19 [28] are two similar networks that only differ in the network depth, i.e., the number of weighted layers—VGG16 has 16 (13 convolutional and three fully connected), and VGG19 has 19 (16 convolutional and three fully connected). These two versions were studied in [28] where they gained more relevance due to their better performance against shallow architectures. ResNets [29] are other deep neural networks that were formulated in 2016 along with the return of the residual functions concept. These neural networks rely on residual learning, i.e., instead of having just a stack of layers (known as plain networks), there are “shortcut” connections that can skip any number of layers. Such connections do not bring extra parameters nor computational complexity to the network [29]. In fact, ResNet proved to be easily optimised and improved, in terms of accuracy, when the depth increases. Three major versions of ResNet were introduced, which differ in the number of layers (depth level): ResNet50 (50 layer ResNet), ResNet101 (101 layer ResNet) and ResNet152 (152 layer ResNet). Among the three, ResNet152 accomplished the lowest error rate on the ImageNet validation set [29]. Additionally, albeit that ResNet152 has a greater depth, being the deepest of the three, it still presents inferior complexity than both other VGGs. Another well-known convolutional neural network is MobileNet [30]. This network was developed for mobile applications and is characterised by its efficiency in latency and size. It provides two hyperparameters that can be tweaked, thus enabling it to fit the model size and speed to the problem requirements [30]. Compared to the VGG topologies, MobileNet is much more lightweight and fast and has almost the same accuracy. There are two new versions of this network: Version 2 (MobileNetV2) added linear bottlenecks and inverted residuals to the depth-wise separable convolutions of the original version, which resulted in even better accuracy, weight and speed [31]; and Version 3 (MobileNetV3) had even better performance in ImageNet classification: MobileNetV3-Large and MobileNetV3-Small attained better accuracy than MobileNetV2, but the large one presented worse latency, while the small showed a similar latency [32].

Over the years, several methods aiming at the recognition of objects in images were proposed, and this work intended to test some of them in order to make a comparative analysis of their performance in the recognition of Tactode tiles.

## 3. Proposed Approach

### 3.1. Image Processing Pipeline

**Figure 2:**
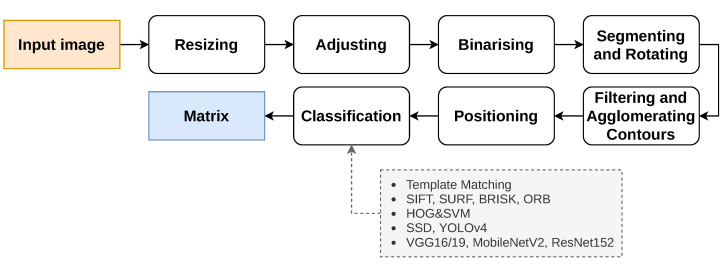
Block diagram of the image processing pipeline.

The image processing pipeline used in this work is common to all classification methods, and its steps are presented in Figure 2. This pipeline reads an image, like the ones presented in Appendix A, and after segmenting it, the tiles are extracted and classified individually. This pipeline is a contribution from a Master of Science thesis [3,4].

**Figure 3:**
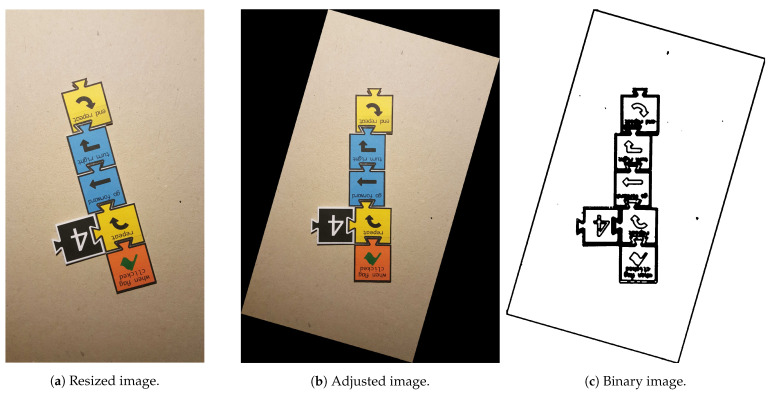

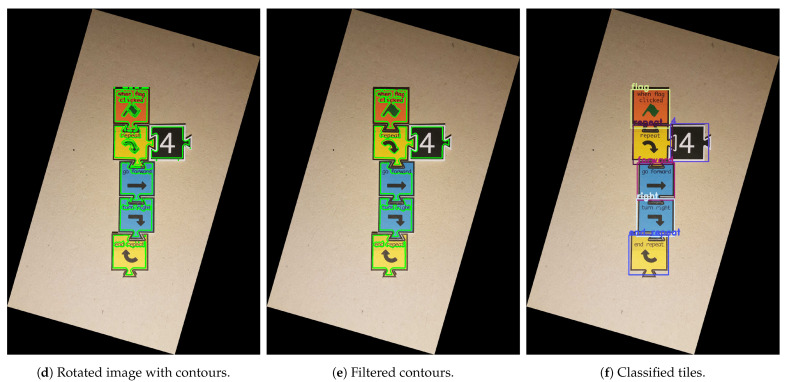
Use case of the processing pipeline: the input image is (**a**) resized, (**b**) adjusted, (**c**) binarised and rotated, and (**d**) the most relevant contours are obtained and (**e**) filtered; then, each contour bounding box is extracted and (**f**) classified.

The pipeline starts by resizing (Figure 3a) the input image in such a fashion that the biggest side (height or width) is resized to 1920 px and the other is resized to a specific dimension, keeping the aspect ratio of the original image. Since the input image can present minimal rotation, it is adjusted by the Hough transform [33] for line detection. This way, the two most important and perpendicular lines are found, and then, the image suffers a small rotation adjustment (Figure 3b) so that both lines reach the vertical or horizontal positions. After, the image is binarised (Figure 3c): an 11 × 11 Gaussian kernel is applied, smoothing the image; a gradient morphological operation is used followed by a colour thresholding, which results in a black and white (binary) image. Furthermore, two additional morphological operations, named opening and closing, help reduce the noise and close possible holes. The three morphological operations are performed with a 6 × 6 cross-structuring element. Next, the binary image is segmented with the methods presented in [34], which extract the contours of the elements present in the image. Moreover, the input image can arrive at this stage rotated 0${}^{\circ }$, 90${}^{\circ }$, 180${}^{\circ }$ or 270${}^{\circ }$ clockwise, since the target users are children, who usually do not care about the rotation of the image. The approach chosen to solve this was based on the detection of “teeth” at the bottom part of each tile bounding box. As can be observed in Figure 1, when the Tactode code is well-oriented, the tiles only have “teeth” on the bottom part or on the right side of their bounding boxes. The tiles that have teeth on the bottom part form a column. Furthermore, the tiles composing the code can be in the column or to its right. Therefore, by checking these two rules—the tiles with detected “teeth” at the bottom have to form a column, and the other tiles have to be in the column or at the right—one can conclude whether the image is rotated or not. Therefore, the image is rotated 90${}^{\circ }$ until the 0${}^{\circ }$ position is reached (when both rules met) or at most four times. The detection of teeth was accomplished by utilising a classifier based on HOG and SVM that was trained by using one of the training datasets, which is detailed in Section 4.1. At this stage, the image is well-oriented, and the pipeline proceeds with the contours’ filtering (Figure 3d,e) in order to get the most interesting ones—the contours that best represent the tiles. Afterwards, each contour bounding box is obtained and positioned in a matrix, which at the end, contains the bounding boxes of the tiles that are placed according to their relative locations in the image. Finally, each bounding box is extracted, adjusted to a square shape, forming ROIs that, in turn, are used to classify the tiles. Such classification is accomplished using methods like the ones that are presented in Section 3.2. The final result is a matrix holding in each position a string-typed variable that represents the name of the tile. As a matter of visualisation, Figure 3f shows a representation of the result generated by the pipeline.

The pipeline was written in JavaScript and Python and was based on OpenCV (Intel Corporation, Willow Garage, Palo Alto, CA, USA) [35]. The JavaScript part uses a Node.js package called opencv4nodejs (GitHub, Inc., San Francisco, CA, USA) [36]. The use of this package was preferred instead of the JavaScript version of OpenCV because the latter does not have the necessary bindings to produce this work. The code is available online (the code repository is available at: https://github.com/daniqsilva25/tactode_recognition, accessed on 30 March 2021).

### 3.2. Classification Methods

The comparative analysis made in this work focused on methods that belong to different areas: neural networks designed to perform object classification or detection; a method that combines image regional descriptors with machine learning; methods based on matching local image features and template matching.

#### 3.2.1. Template Matching

The method based on Template Matching (TM) utilises the template dataset presented in Section 4.1.1 and goes through the following steps: after obtaining the ROI and resizing it to 350 × 350 px, a matching operation is performed between each template and the ROI. Then, the classification of the respective tile is determined by the template that resulted in the largest—in absolute value—maximum or minimum value (depending on the matching mode).

#### 3.2.2. Detection, Description and Matching of Image Features

The methods based on image features’ detection and description also uses the template dataset. The procedure starts by detecting the keypoints and computing the descriptors of a certain ROI, and the same is executed for every template. After, a feature matcher matches the descriptors of the ROI with the descriptors of each template. Then, using RANSAC [37] with a maximum reprojection error threshold of five, the outliers are filtered. Finally, the template that results in more inliers is the one that classifies the respective tile.

The methods for feature detection and description were configured so that the numbers of keypoints detected was similar among them. In addition, the feature matcher used was brute-force along with the L1 norm for SIFT and SURF, since these are float-based descriptors, and the Hamming norm for ORB and BRISK due to the binary nature of their descriptors.

#### 3.2.3. Machine Learning

The method based on machine learning is a combination of HOG and SVM (HOG&SVM), forming a multiclass classifier, since 32 different tiles must be distinguished. Thus, using the training dataset presented in Section 4.1.2, a linear SVM (C = 0.1) was trained using HOG descriptors computed from each data sample. The HOG descriptor was calculated over a window size of 32 × 32 px, divided into cells of 8 × 8 px, with blocks composed by 16 × 16 px (or 2 × 2 cells) and a block stride of 8 × 8 px (or one cell). The gradients were signed, and each histogram had nine bins, leading to a descriptor vector with a length of 324 per sample.

**Figure 4:**
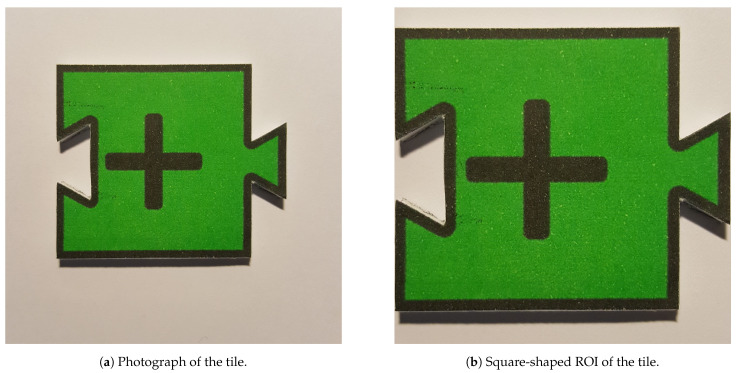
Two images of the tile “addition” for the training the classifier.

During the training, from each image sample, the ROI—part of the image containing the tile—was extracted using the bounding box, which in turn was adjusted to a square shape (1:1 ratio) and resized to 32 × 32 px, respecting the window size of HOG. The descriptors vector was computed and then given to the SVM classifier. Additionally, a 10-fold cross-validation test was performed after the training, which resulted in zero errors. Figure 4 presents two images of the same tile, both related to the ROI extraction operation for training.

With the model already trained, the tiles could be classified after extracting the ROIs, resizing them to 32 × 32 px and using them to compute the HOG descriptors. Then, they were input to the SVM classifier that predicted the labels of the tiles.

In this work, two versions of the HOG&SVM method were developed: a version made on top of OpenCV for Python (HOG&SVM (PY)) and another based on the Node.js package of OpenCV for JavaScript [36] (HOG&SVM (JS)), which was already mentioned. The proceeding sections present some results about both versions, but we focused on the Python version because the other was not an official release.

#### 3.2.4. Deep Learning

The methods based on CNNs for tile classification used in this work were VGG16, VGG19, ResNet152 and MobileNetV2 (MNetV2). The four methods were trained in Google Colaboratory [38], due to its GPU resource availability, with a batch size of 32 and the same training dataset as for the previous method (HOG&SVM). However, in these cases, the ROIs of the training images were resized to 224 × 224 px, as this was the size for which these models were designed. Two important aspects to be noted are the following: the 152 layer version of ResNet was selected due to it being the least error prone [29], and the second version of MobileNet was the primary choice due to the fact that it was the best stable version of MobileNet present in TensorFlow [39], since both versions (large and small) of MobileNetV3 were not yet stable.

To perform the classification of 32 different tiles, the networks were loaded with pre-defined weights (based on the ImageNet dataset classification), and the head of each network was replaced by three new layers sized for the number of classes: a flatten layer, a Rectified Linear Unit (ReLU) activated dense layer and a softmax activated dense layer, which resulted in a final prediction batch with a shape of (32, 32).

After, each base model (with the pre-loaded weights) was frozen in order to keep the original weights unchanged and with the new classification head, they were trained during 20 epochs with a learning rate of 0.0001. Then, some of the top layers of the base models were unfrozen in order to train them, thus fine-tuning the whole model, and again, the model was trained for 20 new epochs with a 10-times lower learning rate (0.00001). At the end, three trained models were obtained, which were exported to be tested later.

In this work, YOLOv4 and SSD-MobileNetV1 (object detectors) were also used as object classifiers, which were trained on the same dataset. Therefore, in this work, both methods received ROIs as the input instead of the entire image.

## 4. Experimental Validation

### 4.1. Datasets

This work produced five datasets: one containing templates of the tiles, two for training the machine learning models and the other two for testing and evaluating the performance, under different metrics, of several classification techniques. All the datasets are available online (the trained models, the datasets and a demonstration video are available at: http://fe.up.pt/asousa/tactode_datasets, accessed on 30 March 2021).

#### 4.1.1. Template Dataset

**Figure 5:**
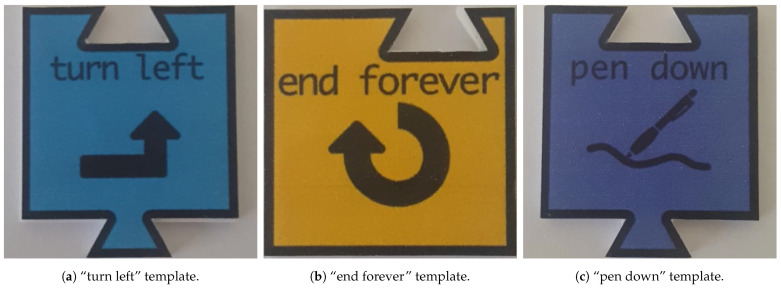
Three templates of the 32 template set.

This dataset contained one image of 350 × 350 px per tile, totalling 32 templates (1 sample/tile × 32 tiles), as the ones shown in Figure 5. This dataset was used by some of the aforementioned classification methods, namely TM and feature matching-based methods.

#### 4.1.2. Training Datasets

The two training datasets were composed of extracted frames from 10 s videos of each Tactode tile that were recorded at 30 fps. The dataset for training a classifier targeting the detection of “teeth” was made up by 2100 positive and 2430 negative samples, and its characteristics are presented in Table 1. The other dataset was made of 9600 images (300 images/tile × 32 tiles) and was created to train the different machine learning classifiers that were mentioned in Section 3.2. Table 2 shows the specifications of this dataset.

#### 4.1.3. Test Datasets

To evaluate the performance of the recognition methods two test datasets were created: *tactode_small* and *tactode_big*. Both datasets had images of different Tactode codes that were gathered using three distinct devices with multiple resolutions. Moreover, all the tests were executed on a laptop running the Ubuntu 18.04 OS with an Intel i7 Quad-core CPU (Intel, Santa Clara, CA, USA) at 2.4GHz and with 4GB of RAM.

**Figure A1:**
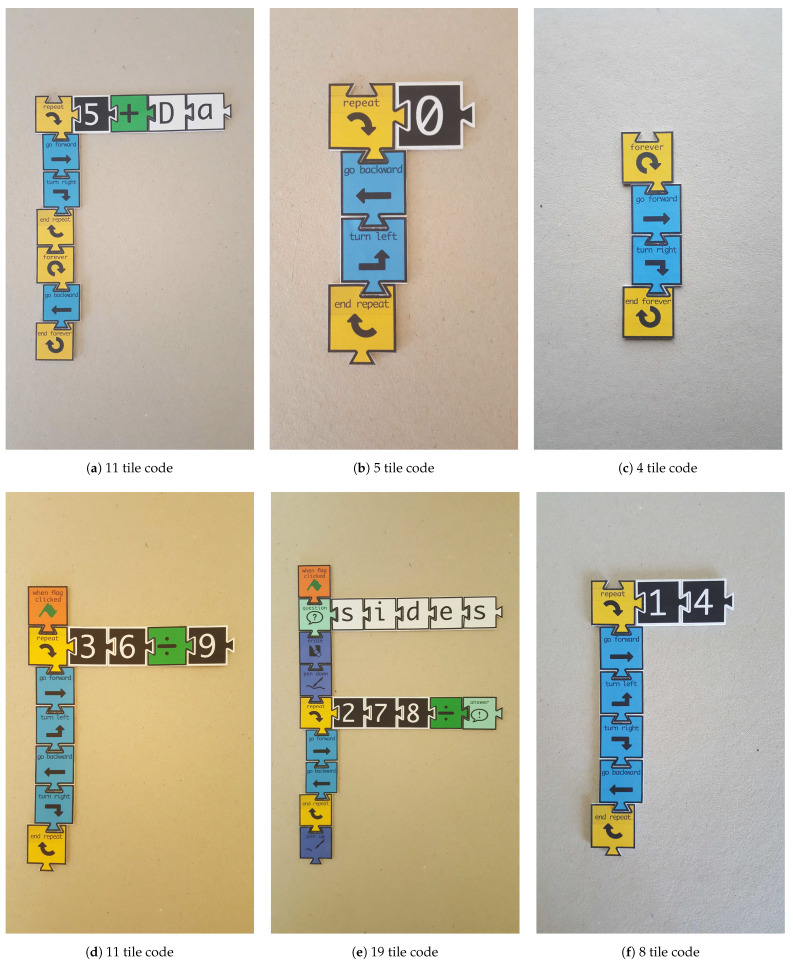
The six Tactode codes that compose the *tactode_small* test dataset.

The *tactode_small* dataset was formed by 288 images and 2784 tiles. The distribution of image resolutions and the number of images and tiles per device is shown in Table 3, where each resolution row of the table has a number of images equivalent to 6 codes × 4 images/code and a number of tiles equal to (11 + 5 + 4 + 11 + 19 + 8) tiles × 4 images. The six codes that this dataset was made of are presented in Figure A1 of Appendix A. In addition, for each Tactode code (set of tiles), there were four images: three were mostly frontal, where two of them presented important rotations (±20${}^{\circ }$); and the other one had a large tilt (the image size of the smallest tile was about 2/3 of the largest one), as can be seen in Figure A3 of Appendix B.

**Figure A2:**
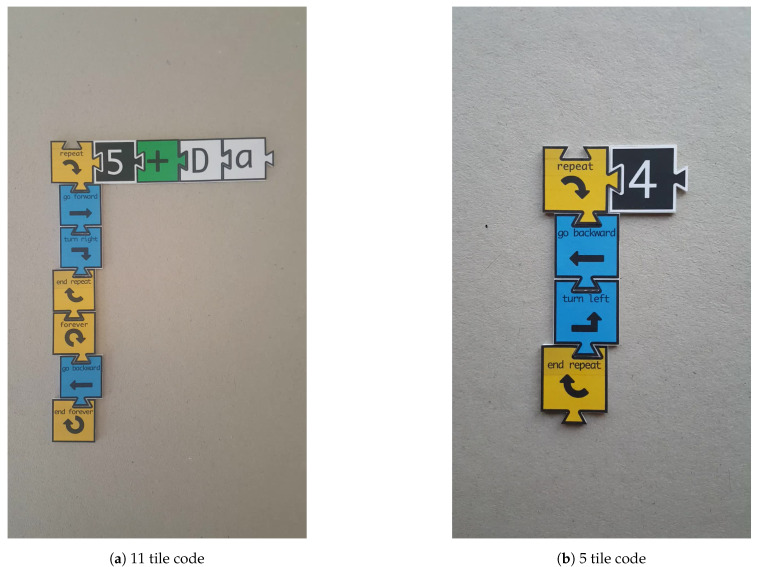
The two Tactode codes that compose the *tactode_big* test dataset.

**Figure A3:**
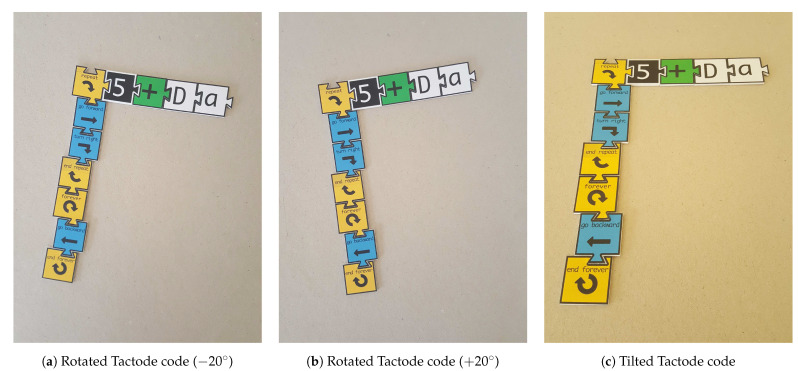
Three Tactode codes demonstrating the rotation (**a**,**b**) and tilt (**c**) present in the datasets.

The *tactode_big* dataset was formed by 12,000 images and 96,000 tiles over two different Tactode codes. These two codes are shown in Figure A2 of Appendix A. All the images corresponded to extracted frames (compressed images) from 10 s videos that were taped at 30 fps for each code. In addition, each code was recorded presenting four rotation angles—0${}^{\circ }$, 90${}^{\circ }$, 180${}^{\circ }$ and 270${}^{\circ }$ clockwise—giving one video per angle, thus resulting in rotated test images. The distribution of resolutions, rotation angles and the number of images and tiles per device is shown in Table 4, where each resolution row of the table has a number of images equivalent to 2 codes × 300 images/code and a number of tiles equivalent to (11 + 5) tiles × 300 images.

### 4.2. Evaluation Metrics

In this section, we focus on assessing the classification errors introduced by the tests. Therefore, a metric called accuracy was used and is defined by (1), where *T* and MT represent the number of tiles and misclassified tiles, respectively.

(1) $$Accuracy=\frac{T-MT}{T}\times 100\%$$

However, it was necessary to add a variation of the accuracy metric in the case that any of the methods resulted in some unclassified tiles, mainly the object detectors (YOLO and SSD) that sometimes could not localise any object (tile) inside an ROI. Therefore, this version of the accuracy was accomplished by (2) and differed from (1) in the aspect that the total number of tiles (*T*) was subtracted by the number of unclassified tiles (UT). This way, the accuracy of the methods that originated tiles without classification was measured more fairly.

(2) $$Accuracy^{*}=\frac{(T-UT)-MT}{T-UT}\times 100\%$$

Additionally, the Execution Time Mean Value (ETMV), Execution Time Standard Deviation (ETSD), minimum Execution Time (minET) and maximum Execution Time (maxET) were also measured to evaluate the computational cost of the methods. Such time metrics are defined in (3)–(), where *N* is the number of executions, $t_{i}$ is the execution time at instance *i*, μ is the mean value, σ is the standard deviation and min and max are the minimum and maximum values of the execution times, respectively.

(3) $$\begin{array}{c} \mu =\frac{\sum_{t=1}^{N}t_{i}}{N} \end{array}$$

(4) $$\begin{array}{c} \sigma =\sqrt{\frac{\sum_{t=1}^{N}{\left(t_{i}-\mu \right)}^{2}}{N}} \end{array}$$

(5) $$\begin{array}{c} min=argmin(t_{1},t_{2},...,t_{N}) \end{array}$$

(6) $$\begin{array}{c} max=argmax(t_{1},t_{2},...,t_{N}) \end{array}$$

## 5. Results

In this section, the results of the tests performed on the two test datasets are presented, and they are analysed and discussed later on in Section 6.

The results of the tests executed on the *tactode_small* dataset are exposed in Table 5 and Table 6. For each method, Table 5 shows the accuracy results globally and across all available resolutions, and Table 6 presents the results concerning their execution time. A detail that must be mentioned is that the resolutions of 6 and 8 Mpx had twice the number of images as the other resolutions, since two of the devices that were used had both of these resolutions available.

An issue that can be observed in Table 5 is the decrease in accuracy with increasing resolution in some column transitions, for instance from Column 3.1 to 3.7, where a general decrease in performance can be easily noted. This problem is discussed later in Section 6.

The tests executed on the *tactode_big* dataset were only done for VGG16, VGG19, ResNet152, MNetV2 and HOG&SVM because the previous results (obtained from the *tactode_small* dataset) showed that these were the best classification methods. Therefore, focusing only on these methods, the results acquired from the tests executed on the *tactode_big* dataset are presented in Table 7 and Table 8. For each of the five methods, Table 7 exhibits the accuracy results globally and across all available resolutions, and Table 8 shows the results concerning their execution time. It is important to mention that: the resolutions of 3.7 and 8.3 Mpx contained 19,200 tiles each, while the 2.1 Mpx resolution contained 57,600 tiles; and VGG16, VGG19, ResNet152 and MNetV2 were tested with Google Colaboratory due to the easy access to GPU power.

## 6. Comparative Analysis

**Figure 6:**
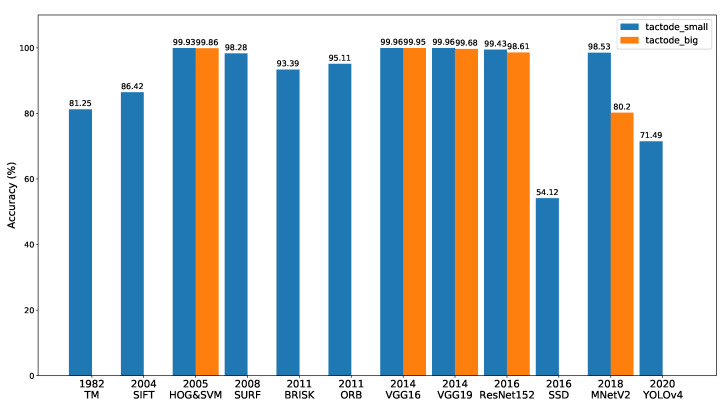
Timeline-like representation of the results obtained over the two test datasets with the global accuracy of all recognition methods.

In Figure 6, a bar graph is shown that represents the performance of all methods on the two test datasets and ordered by the year of the appearance of the methods. This figure is mentioned throughout this section to make a comparative analysis among the recognition methods.

The results obtained from *tactode_small* are exposed in Table 5 and Table 6, where it can be noticed that both versions of the TM-based method attained reasonable accuracy values (81.25% and 69.47% for the maximum and minimum version, respectively), considering that this method is the oldest one, according to Figure 6. Furthermore, they reached average execution times of 2.673 s and 3.495 s.

Regarding the methods based on image features matching, SURF achieved the best accuracy (98.28%), and ORB and BRISK were second and third with an accuracy of 95.11% and 93.39%, respectively. On the other hand, SIFT was the one, among this type of methods, that presented more classification errors and a resulting accuracy of 86.42%. The reason behind this is that SIFT is more sensitive to noise than, for instance, SURF [11] because the latter uses a sub-patch to compose the gradient information, whereas SIFT depends on individual gradients’ orientations. This makes SURF, with the use of a global descriptor, more robust to image perturbations than SIFT, which, in turn, relies on a local descriptor. Those perturbations can be light condition variations, which, as a matter of fact, appeared to be present in this work, as shown in Figure A1, where all the images share the same background, yet some colour variations of the same in the images can be seen. This detail compromised the performance of SIFT since the pair template-ROI can potentially exhibit such variations. In terms of temporal execution, ORB was the fastest with an average execution time of 9.261 s, followed by BRISK with 12.637 s. SIFT presented an average of 17.810 s of execution, and SURF, which typically has less computational cost than SIFT [11], was overall the worst method on this parameter, taking on average 36.720 s to execute. This was due to the fact that SURF detects more keypoints than SIFT [40], therefore resulting in more outliers, and consequently, the outliers filtering phase slowed down SURF in the classification stage.

With respect to the object detectors (SSD and YOLOv4), both presented poor results (54.12% for SSD and 71.49% for YOLOv4) possibly because the training dataset was not the most suitable for them to detect Tactode tiles, since the test images were composed by Tactode codes (connected tiles), while the training images contained only individual tiles; it was expected that with an adequate training dataset, their performance could be improved.

HOG&SVM presented an accuracy of 99.93% and 99.86% along with an average execution time of 0.323 s and 0.232 s on *tactode_small* and *tactode_big*, respectively. Besides, it is important to mention that the two versions of HOG&SVM (Python and JavaScript) generated different results due to the fact that the implementation of this method was different in the OpenCV library for Python from the OpenCV Node.js package.

As regards the CNN-based classifiers, VGG16 and VGG19 were the best performers in terms of accuracy on the *tactode_small* test dataset with an accuracy tie of 99.96% and an average execution time of 3.369 s and 4.479 s, respectively. On the same dataset, ResNet152 achieved an accuracy of 99.43% with an average execution time of 3.740 s, and MNetV2 obtained a 98.53% accuracy and a 0.920 s average execution time. On the *tactode_big* test dataset, VGG16 proved to be the best method, reaching an accuracy of 99.95%, followed by VGG19 with 99.68%, ResNet152 with 98.61% and MNetV2 with 80.20%. On this bigger dataset, this type of method presented lower execution times by the use of the GPU; thus, the temporal values of 0.583 s, 0.621 s, 0.791 s and 0.628 s for VGG16, VGG19, ResNet152 and MNetV2, respectively, were not comparable to those of HOG&SVM because this was tested on a CPU for both test datasets.

In Figure 6, from *tactode_small* and *tactode_big*, it can be noticed that the accuracy of HOG&SVM, ResNet152, MNetV2 and both VGGs decreased. We believed that this phenomenon was related to the fact that the images of *tactode_big* dataset were obtained by extracting the frames from videos at 30 fps that generally underwent compression processes, hence originating quality losses in the extracted images. MNetV2 appeared to be the method where this decrease in accuracy was more obvious: its accuracy dropped from 98.53% to 80.20%. Additionally, the resolution that contributed the most to this drop was the smallest one available for *tactode_big*, which was 1920 × 1080 px (2.1 Mpx) with a 72.05% accuracy, as can be seen in Table 7. It was expected that MNetV2 would be worse than both VGGs and ResNet152 since it was designed targeting mobile applications, where memory efficiency and temporal performance are key characteristics [31], which in fact can be verified in this case, whereas MNetV2 resulted in the third best average execution time on *tactode_small* (tested on CPU). The accuracy results of VGG16 and VGG19 were similar, as was expected [28], and their execution times were in accord with the expectations, because VGG16 had fewer layers than VGG19 (16 against 19 layers) and presented a lower computational time than the latter on both test datasets. With respect to HOG&SVM, it presented a minimal difference in accuracy compared to both VGG CNNs on *tactode_small* and VGG16 in *tactode_big*, at the same time, being the fastest overall method on both datasets. This worse accuracy in comparison to the VGGs could be derived from HOG&SVM using an input image of 32 × 32 px, while both VGGs used a 224 × 224 px image. Such a resolution difference could have an impact on performance since, in the case of HOG&SVM, there was less available information (pixels) to use for tile recognition. In spite of that, the results of HOG&SVM were impressive considering that this method was nine years older than VGG, as can be noticed in Figure 6. With respect to ResNet152, we suspected that the abrupt difference of the maximum execution time that this method presented during the tests on the *tactode_big* dataset compared to the other CNN-based methods (3.108 s against ≈ 1 s) was derived from the fact that ResNet152 was memory-wise the largest model, thus requiring more time to load before performing any classification. In fact, this was an important aspect concerning the execution time of the methods, since it was expected that methods that were more computationally complex or required more memory space (for instance, VGG16, VGG19, ResNet152 or YOLO) took a longer time to perform computations than lighter and simpler methods (such as HOG&SVM, MNetV2 or SSD).

Concerning the issue raised in Table 5 about the decrease in performance with the increase in resolution, we believed that this problem may originate from the segmentation step of the pipeline, specifically with the contours’ extraction precision, because sometimes, the bounding boxes of the contours did not cover the tiles entirely, and this could potentially interfere with the recognition performance of the methods.

After analysing the results exposed in Section 5, it can be said that VGG16 was the best recognition method in terms of accuracy. However, when the trade-off between accuracy and computing time is at stake and a GPU is not available, HOG&SVM would be the best choice.

## 7. Conclusions

The motivation for this work was Tactode tiles’ recognition within a 2D scene. In the Tactode application, a large number of interconnected tiles is present. Each tile must be detected and recognised by shape and content. The main focus of this work was related to 2D object recognition, resulting in an in-depth comparative analysis of 12 well-known methods (Figure 6). The study benchmarked the accuracy and execution time of these methods by using two public datasets: *tactode_small* and *tactode_big*.

The studied methods can be grouped into four categories: (i) classic template matching, (ii) methods based on handcrafted features, (iii) machine learning with HOG and SVM and (iv) deep learning with CNN-based methods. The first category is template matching featuring an accuracy of 81.25%. The best performer in the second category was SURF with about 98% accuracy. In this category, the worst method was SIFT due to its high sensitivity to luminosity and colour variations. Interestingly, HOG&SVM (third category) was the best overall method in terms of execution time, reaching average times of 0.323 s on *tactode_small* and 0.232 s on *tactode_big*, with accuracies above 99.8% on both datasets. From the tested methods in the fourth category, VGG16 proved to be the best in terms of accuracy (above 99.9% on both datasets).

If accuracy is the utmost concern, then VGG16 is the best choice for recognition tasks, but for an application with low computational resources or under stringent real-time constraints, HOG&SVM is very likely to be the best method because of its reduced execution time with a slight loss in accuracy. Additionally, the integration of HOG&SVM with a web application such as the Tactode web application would be easier due to the availability of JavaScript source code.

The presented study is relevant for applications involving 2D object recognition, in particular those applications that take advantage of the identification of a substantial number of interconnected and repeated objects in the scene. Additionally, the presented comparative analysis is relevant in applications where resource usage and time constraints are at stake.

Future work includes extending the tile set with other Tactode tiles and re-evaluating the performance of the recognition methods with the addition of new classes to the problem at hand, as well as building an adequate dataset for training the object detectors (YOLO and SSD) correctly, in order to improve their performance.

## Figures and Tables

**Table 1:** Specifications of the training dataset for the detection of tiles “teeth”.

Resolution (Mpx); [Height × Width] (px)	Type of Sample	Number of Images
8.3; [3840 × 2160]	positive (with “teeth”)	2100
	negative (without “teeth”)	2430

**Table 2:** Specifications of the training dataset for the classification of tiles.

Resolution (Mpx); [Height × Width] (px)	Number of Images
8.3; [3840 × 2160]	9600

**Table 3:** Specifications of the *tactode_small* test dataset.

Device	Resolution (Mpx); [Height × Width] (px)	Number of Images	Number of Tiles
Samsung Galaxy S6	16; [5312 × 2988]	24	232
	12; [3984 × 2988]	24	232
	8.9; [2976 × 2976]	24	232
	8.0; [3264 × 2448]	24	232
	6.0; [3264 × 1836]	24	232
Huawei P Smart+	10; [3648 × 2736]	24	232
Samsung Galaxy Tab S	8.0; [3264 × 2448]	24	232
	6.0; [3264 × 1836]	24	232
	4.9; [2560 × 1920]	24	232
	3.7; [2560 × 1440]	24	232
	3.1; [2048 × 1536]	24	232
	2.4; [2048 × 1152]	24	232
Total		288	2784

**Table 4:** Specifications of the *tactode_big* test dataset.

Device	Resolution (Mpx); [Height × Width] (px)	Rotation Angle (Clockwise)	Number of Images	Number of Tiles
Samsung Galaxy S6	8.3; [3840 × 2160]	0${}^{\circ }$	600	4800
		90${}^{\circ }$	600	4800
		180${}^{\circ }$	600	4800
		270${}^{\circ }$	600	4800
	3.7; [2560 × 1440]	0${}^{\circ }$	600	4800
		90${}^{\circ }$	600	4800
		180${}^{\circ }$	600	4800
		270${}^{\circ }$	600	4800
	2.1; [1920 × 1080]	0${}^{\circ }$	600	4800
		90${}^{\circ }$	600	4800
		180${}^{\circ }$	600	4800
		270${}^{\circ }$	600	4800
Huawei P Smart+	2.1; [1920 × 1080]	0${}^{\circ }$	600	4800
		90${}^{\circ }$	600	4800
		180${}^{\circ }$	600	4800
		270${}^{\circ }$	600	4800
Samsung Galaxy Tab S	2.1; [1920 × 1080]	0${}^{\circ }$	600	4800
		90${}^{\circ }$	600	4800
		180${}^{\circ }$	600	4800
		270${}^{\circ }$	600	4800
Total			12,000	96,000

**Table 5:** Accuracy results of the tests performed on the *tactode_small* test dataset and their distribution by the available resolutions. The “*” in YOLOv4 and SSD indicates that their accuracy values were computed according to Equation (2). JS, JavaScript; PY, Python.

Method	Accuracy (%)										
	Global	Resolution (Mpx)									
		2.4	3.1	3.7	4.9	6	8	8.9	10	12	16
VGG16	99.96	99.57	100	100	100	100	100	100	100	100	100
VGG19	99.96	100	100	100	100	100	100	100	99.57	100	100
ResNet152	99.43	99.14	98.71	98.71	99.14	99.57	100	100	98.71	99.57	100
MNetV2	98.53	95.69	98.71	96.55	98.28	98.28	99.78	100	97.84	99.57	99.57
YOLOv4 *	71.49	70.89	74.18	75.00	68.12	71.29	71.71	65.83	70.62	74.27	72.68
SSD *	54.12	78.85	77.19	58.30	60.26	46.36	48.98	39.82	61.79	38.71	42.48
HOG&SVM (JS)	100	100	100	100	100	100	100	100	100	100	100
HOG&SVM (PY)	99.93	100	100	100	100	100	100	100	99.14	100	100
SURF	98.28	98.28	99.14	96.98	98.71	96.77	98.92	99.14	99.14	99.14	97.41
ORB	95.11	94.83	96.55	95.69	95.26	95.04	94.40	94.83	95.69	95.69	93.97
BRISK	93.39	90.52	94.40	87.93	94.40	92.03	94.83	93.97	96.12	94.83	94.83
SIFT	86.42	85.78	84.91	84.48	84.91	85.34	88.15	88.79	89.22	87.50	84.48
TM (max)	81.25	66.81	74.14	83.19	88.79	83.19	85.56	87.07	67.67	84.05	85.78
TM (min)	69.47	64.22	67.67	82.33	87.07	69.18	70.91	63.79	62.93	62.50	62.93

**Table 6:** Temporal results of the tests performed on the *tactode_small* test dataset.

Method	ETMV (s)	ETSD (s)	minET (s)	maxET (s)
VGG16	3.639	1.756	1.333	7.889
VGG19	4.479	2.174	1.710	9.913
ResNet152	3.740	1.760	1.554	7.812
MNetV2	0.920	0.327	0.422	1.816
YOLOv4	10.284	5.312	3.847	22.572
SSD	0.872	0.329	0.418	2.528
HOG&SVM (JS)	0.228	0.054	0.135	0.424
HOG&SVM (PY)	0.323	0.073	0.207	0.518
SURF	36.720	18.549	15.017	73.661
ORB	9.261	4.224	3.553	20.953
BRISK	12.637	6.082	5.194	31.057
SIFT	17.810	8.445	7.194	42.051
TM (max)	2.673	1.256	1.066	5.947
TM (min)	3.495	1.631	1.512	6.783

**Table 7:** Accuracy results of the tests performed on the *tactode_big* test dataset and their distribution by the available resolutions.

Method	Accuracy (%)			
	Global	Resolution (Mpx)		
		2.1	3.7	8.3
VGG16	99.95	99.91	99.99	100
VGG19	99.68	99.50	99.90	100
ResNet152	98.61	98.08	98.88	99.95
MNetV2	80.20	72.05	91.31	93.56
HOG&SVM (JS)	99.99	99.98	100	100
HOG&SVM (PY)	99.86	99.81	99.96	99.92

**Table 8:** Temporal results of the tests performed on the *tactode_big* test dataset.

Method	ETMV (s)	ETSD (s)	minET (s)	maxET (s)
VGG16	0.583	0.104	0.409	1.173
VGG19	0.621	0.107	0.452	1.128
ResNet152	0.791	0.165	0.542	3.108
MNetV2	0.628	0.108	0.424	1.326
HOG&SVM (JS)	0.162	0.041	0.094	0.422
HOG&SVM (PY)	0.232	0.035	0.148	0.415

## Data Availability

The datasets generated during this work, the trained models and a demonstration video are publicly available at http://fe.up.pt/asousa/tactode_datasets (accessed on 30 March 2021).

## Abbreviations

The following abbreviations are used in this paper:

BRIEF	Binary Robust Independent Elementary Features
BRISK	Binary Robust Invariant Scalable Keypoints
CNN	Convolutional Neural Network
CPU	Central Processing Unit
ETMV	Execution Time Mean Value
ETSD	Execution Time Standard Deviation
FAST	Feature from Accelerated Segment Test
GPU	Graphical Processing Unit
HOG	Histograms of Oriented Gradients
HOG&SVM	HOG and SVM
maxET	maximum Execution Time
minET	minimum Execution Time
MNetV2	MobileNetV2
ORB	Oriented FAST and Rotated BRIEF
OS	Operating System
RAM	Random Access Memory
RANSAC	Random Sample Consensus
ReLU	Rectified Linear Unit
ROI	Region Of Interest
SIFT	Scale-Invariant Feature Transform
SSD	Single-Shot Multibox Detector
SURF	Speeded-Up Robust Features
SVM	Support Vector Machine
TM	Template Matching
YOLO	You Only Look Once
